# Adequação das Práticas do Laboratório de Cateterismo durante a Pandemia de COVID-19: O Protocolo do Instituto Dante Pazzanese de Cardiologia

**DOI:** 10.36660/abc.20200489

**Published:** 2020-09-18

**Authors:** Daniel Chamié, Fernanda Oliveira, Sérgio Braga, José Ribamar Costa, Dimytri Alexandre Alvim de Siqueira, Rodolfo Staico, Ricardo Costa, Galo Maldonado, Luiz Fernando Leite Tanajura, Marinella Patrizia Centemero, Áurea Jacob Chaves, Andrea Cláudia Sousa Leão Abizaid, Rafaela Andrade Penalva Freitas, Nancy Toledo Coelho, Louis Nakayama Ohe, Cely Abboud, Fausto Feres

**Affiliations:** 1 Instituto Dante Pazzanese de Cardiologia São Paulo SP Brasil Instituto Dante Pazzanese de Cardiologia , São Paulo , SP – Brasil

**Keywords:** Betacoronavírus/complicações, Betacoronavírus/epidemiologia, Doenças Cardiovasculares/complicações, Infecções por Coronavírus, Restruturação Hospitalar, Guia de Prática Clínica, Habilidade para Realização de Testes

## Introdução

A pandemia global da doença de coronavírus 2019 (COVID-19) causada pelo novo vírus de síndrome respiratória aguda grave coronavírus 2 (SARS-CoV-2) começou em Wuhan, China, em dezembro de 2019 e afetou mais de 4,4 milhões de pessoas em todo o mundo, com 302.169 mortes até o dia 16 de maio de 2020. ^1^

Embora os sintomas respiratórios sejam a apresentação mais comum de COVID-19, o envolvimento cardíaco é uma característica proeminente dessa doença, ocorrendo em 20% a 30% dos pacientes hospitalizados e contribuindo para 40% dos óbitos. ^2 - 4^ O envolvimento cardíaco relacionado à COVID-19 tem sido documentado por elevações em biomarcadores cardíacos e frequentemente apresenta alterações no segmento ST-T no eletrocardiograma (ECG) de 12 derivações, motivo pelo qual a equipe do laboratório de cateterismo é frequentemente ativada. Além disso, as atividades do laboratório de cateterismo devem continuar no atendimento a pacientes não COVID-19 que apresentam síndrome coronariana aguda (SCA) verdadeira, infarto do miocárdio com supradesnivelamento do segmento ST (IAMCSST) e doença cardíaca isquêmica estável muito sintomática.

Devido à escalada no número de casos de COVID-19 na cidade de São Paulo, epicentro da doença no Brasil, reformularam-se a logística e as práticas no laboratório de cateterismo cardíaco do Instituto Dante Pazzanese de Cardiologia, que entraram em vigor em abril de 2020 e continuarão durante o período da pandemia. Os objetivos são fornecer atendimento otimizado à população que necessita de procedimentos cardíacos invasivos durante a pandemia, com a proteção adequada aos profissionais de saúde (PS), pacientes e seus familiares.

Os protocolos aqui descritos representam os esforços multidisciplinares e dinâmicos do Departamento de Cardiologia Invasiva do Instituto Dante Pazzanese de Cardiologia validados pelo Comitê de Controle de Infecção da instituição. Essas práticas estão sujeitas a alterações em função do estado epidemiológico local, a fase da epidemia e a disponibilidade de equipamento de proteção individual (EPI). Estes protocolos podem não se aplicar a outras localidades sem casos (ou casos esporádicos) de COVID-19 ou a serviços que atendem diferentes perfis populacionais com logísticas e disponibilidade de EPI diversas.

### Epidemiologia e Transmissão de SARS-CoV-2 e Justificativa para Reestruturação das Práticas

As informações atuais sugerem que as principais vias de transmissão de SARS-CoV-2 são por meio de gotículas respiratórias e contato direto e indireto com superfícies contaminadas. O tamanho de aerossol exalado depende das características do fluido, da força e da pressão no momento da emissão e das condições ambientais. Partículas grandes permanecem suspensas no ar por um período curto e se depositam a menos de 1 metro da fonte. As partículas menores evaporam rapidamente, enquanto os resíduos secos se assentam lentamente e permanecem suspensos por um período variável de tempo. Os aerossóis respiratórios infecciosos são 1) gotículas (aerossol respiratório > 5 μm de diâmetro) e 2) núcleos de gotículas [a parte seca do aerossol (< 5 μm de diâmetro)], que resultam da evaporação de gotículas tossidas ou espirradas ou de partículas infecciosas exaladas. A proximidade habitual entre as pessoas é suficiente para que haja transmissão de vírus. ^5^ Há uma probabilidade substancial de que a fala normal cause a transmissão do vírus no ar em ambientes fechados. ^6^ Tossir e espirrar podem propagar nuvens de aerossóis até 7 a 8 metros (23 a 26 pés) ^7^ Gotículas também podem pousar em superfícies onde o vírus pode permanecer viável, servindo como fonte de transmissão de contato. O SARS-CoV-2 pode permanecer estável na forma aerossolizada por até 3 horas, em materiais de papelão até 24 horas e em superfícies plásticas ou metálicas até 3 dias. ^8^

O período médio de incubação é de 5,2 dias (intervalo de confiança de 95%: 4,1 a 7,0 dias), com o percentil 95 da distribuição em 12,5 dias. ^9 , 10^ O R0 (R zero, número básico de reprodução) estimado da SARS-CoV-2 está entre 2 e 3, o que significa que cada pessoa com SARS-CoV-2 infectará 2 a 3 outras pessoas dentro de uma população suscetível. ^10 , 11^ A carga viral detectada é semelhante em pacientes com COVID-19 assintomáticos e sintomáticos, o que sugere que o vírus pode ser potencialmente transmitido de pacientes assintomáticos ou minimamente sintomáticos. ^12^ As estimativas indicam que 86% de todas as infecções não foram documentadas. É importante que estas foram a fonte de infecção de 79% dos casos documentados. ^13^

PS estão em maior risco de infecção. Um relatório recente sobre 138 casos confirmados de COVID-19 revelou que 41,3% foram adquiridos no hospital, 70% dos quais eram PS. ^14^ Os procedimentos geradores de aerossóis acarretam maior risco de transmissão aérea do vírus aos PS. Entre estes, estão a intubação traqueal, a ventilação não invasiva, a traqueotomia, a ressuscitação cardiopulmonar, a ventilação manual antes da intubação, a broncoscopia e os procedimentos odontológicos, oftalmológicos e otorrinolaringológicos. ^15^

Os vírus respiratórios geralmente não são transmitidos pelo sangue. ^16^ Assim, no caso específico de PS no laboratório de cateterismo, o maior risco de exposição ao SARS-CoV-2 está relacionado à proximidade com os pacientes e ao contato com superfícies contaminadas, por exemplo, em campos cirúrgicos, aventais e equipamentos.

Idealmente, todos os pacientes deveriam ser testados para SARS-CoV-2 antes de entrar no laboratório de cateterismo cardíaco. Exceções a isso são, talvez, os pacientes encaminhados para procedimentos de emergência (e.g. pacientes com IAMCSST), pelos quais não há tempo para aguardar os resultados dos exames; estes devem ser tratados como pacientes com COVID-19 confirmado. Atualmente, no Brasil, os testes não estão amplamente disponíveis, e os resultados não estão saindo em tempo hábil. Portanto, é intuitivo que o uso universal de EPI completo seja desejável. No entanto a escassez de EPI é uma realidade em todo o mundo e o uso adequado e racional de equipamentos de proteção é necessário para otimizar a sua disponibilidade.

Como resultado, várias estratégias têm sido implementadas para proporcionar proteção adequada a todos os PS e aos pacientes sem deixar de garantir um atendimento de qualidade aos pacientes.

### Ajustes Logísticos Adotados antes da Entrada de Pacientes no Laboratório de Cateterismo

**Figura 1:**
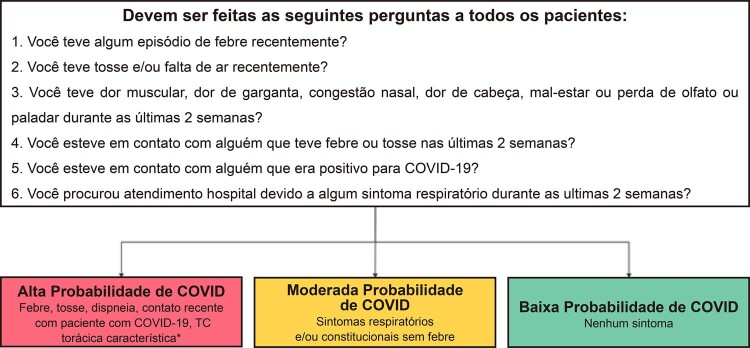
– Questionário de triagem para pacientes encaminhados para procedimentos cardíacos invasivos. TC: tomografia computadorizada. * TC torácica característica foi definida como opacidades em vidro fosco com distribuição periférica e basal predominante.

- Foram realizados ajustes na agenda para minimizar os tempos de espera na área de recepção do Departamento de Cardiologia Invasiva;
- É permitido apenas um membro da família ou acompanhante por paciente para minimizar o número de pessoas circulando no Departamento de Cardiologia Invasiva;
- É obrigatório o uso de máscara cirúrgica por todos os pacientes e seus acompanhantes durante todo o tempo que estiverem dentro do hospital;
- A equipe médica e de enfermagem deve usar máscara cirúrgica ou respiradores FFP2/N95 (conforme apropriado) durante o contato com os pacientes e realizar a higiene das mãos após cada contato;
- Os pacientes encaminhados do departamento de emergência (DE) ou pacientes internados encaminhados de outras instituições são considerados casos suspeitos de COVID-19 e o EPI completo deve ser usado;
- No momento da chegada, temperatura deve ser medida em todos os pacientes com termômetro infra-vermelho sem contato com os mesmos. Pacientes com temperatura ≥ 37,0 ºC terão seus procedimentos adiados e serão encaminhados para uma área destinada a pacientes com suspeita de COVID-19;
- A saturação de oxigênio deve ser obtida em todos os pacientes usando oxímetro de pulso de dedo no momento da chegada. Pacientes com saturação de oxigênio < 94% terão seus procedimentos adiados e serão encaminhados para área destinada a pacientes com suspeita de COVID-19. Deve-se prestar atenção à identificação de outras condições médicas associadas à baixa saturação de oxigênio (e.g. insuficiência cardíaca descompensada, doença pulmonar crônica)
- Considerando que o uso universal de EPI não é uma realidade neste momento, é necessário tentar identificar os pacientes que apresentam maior risco de transmissão de infecção. Embora a transmissão de SARS-CoV-2 possa ocorrer na fase pré-sintomática, tem sido demonstrado que as cargas virais nos *swabs* de garganta e nas amostras de escarro atingiram o pico em cerca de 5 a 6 dias após o início dos sintomas. ^17^ Portanto, um questionário dedicado está sendo aplicado a todos os pacientes em relação ao seu estado sintomático e aos seus contatos com pacientes com COVID-19. A COVID-19 causa uma série de sintomas constitucionais não específicos, sintomas respiratórios superiores e inferiores e, menos frequentemente, sintomas gastrointestinais. Os três principais sintomas do COVID-19 são a febre, a tosse e a falta de ar. Outros sintomas relatados incluem mialgia, anorexia, mal-estar, dor de garganta, congestão nasal, cefaleia e nova perda de paladar ou olfato. Os sintomas podem aparecer em apenas 2 dias ou em até 14 dias após a exposição. ^18^ Os pacientes são, subsequentemente, estratificados por risco quanto à probabilidade de COVID-19, conforme mostrado na Figura 1.
- O EPI completo deve ser usado por todos os PS envolvidos em procedimentos com pacientes categorizados como amarelo (probabilidade moderada de COVID) ou vermelho (probabilidade alta de COVID).

### Ajustes Logísticos nas Práticas de Rotina do Laboratório de Cateterismo

Na preparação para cuidar de pacientes com COVID-19, deve-se garantir que os pacientes sem COVID-19 que precisam de cuidados cardiovasculares invasivos continuem recebendo atendimento em um ambiente seguro. Portanto, têm sido implementados ajustes contínuos na prática da nossa unidade de cardiologia invasiva. Adaptações no protocolo serão determinadas principalmente pela disponibilidade de testes para SARS-CoV-2, suprimento de EPI, e o estado de saúde da força de trabalho. Descrevemos a seguir uma lista das principais mudanças nas práticas de nosso departamento.

- A unidade de atendimento diário do laboratório de cateterismo cardíaco de 20 leitos está localizada perto e no mesmo andar dos laboratórios de cateterismo. Esta área recebe pacientes provenientes de seus domicílios, ou encaminhados de outros hospitais, para preparo para realização de cateterismos cardíacos diagnósticos, intervenções coronárias percutâneas (ICP), procedimentos endovasculares e de doenças cardíacas congênitas. Os pacientes são atendidos por uma extensa equipe de enfermeiros, cardiologistas e *fellows* de primeiro ano em cardiologia. Cuidados pós-procedimentos também são oferecidos nessa área. Durante a pandemia de COVID-19, a metade desta unidade tem sido dedicada aos pacientes com suspeita de COVID-19 (zona amarela) e a outra metade aos pacientes sem COVID-19 ou com baixa probabilidade de COVID-19 (zona verde). Essas duas zonas foram fisicamente separadas. A equipe de saúde também foi dividida para trabalhar nas duas áreas de forma independente. Aqueles que cuidam de pacientes com suspeita de COVID-19 não cuidam do setor negativo de COVID-19 e vice-versa. O EPI adequado está disponível nos dois setores.
- Os pacientes ambulatoriais com suspeita de COVID-19 e os pacientes provenientes do DE, da unidade de terapia intensiva (UTI) ou da área de COVID-19 do hospital entram e saem dos laboratórios de cateterismo cardíaco por rotas específicas que são diferentes daquelas utilizadas por pacientes não COVID.
- Do total de seis laboratórios de cateterismo, dois têm sido dedicados a pacientes positivos ou suspeitos de COVID-19. No caso de o número de pacientes suspeitos de COVID exceder o número de pacientes não COVID, duas salas adicionais podem ser convertidas em laboratórios de COVID. Estamos estruturados para operar quatro laboratórios COVID e dois não COVID simultaneamente.
- As macas usadas para levar pacientes positivos ou suspeitos de COVID-19 ao laboratório de cateterismo são mantidas dentro do laboratório para minimizar a exposição às outras áreas do Departamento de Intervenção.
- Com os objetivos de reduzir o número de pessoal circulante e racionar os EPI, o pessoal dos laboratórios de COVID tem sido limitado ao mínimo, todos usando EPI completo.
- As portas dos laboratórios de COVID devem permanecer fechadas em todos momentos.
- Nenhum dos nossos laboratórios de cateterismo está equipado com sistema de pressão negativa. Por isso, cada laboratório de COVID passa por limpeza terminal após cada procedimento

### Equipamentos de Proteção para Profissionais de Saúde

**Figura 2:**
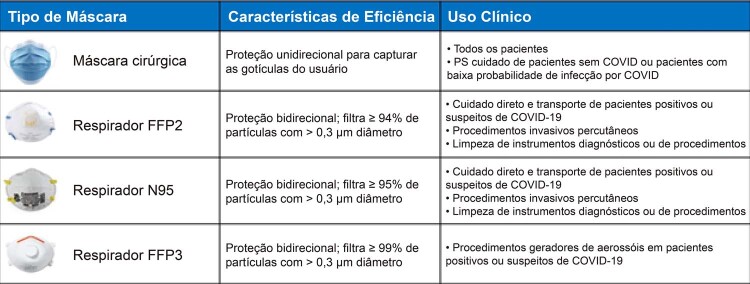
– Tipos de máscaras/respiradores de acordo com a eficácia de filtragem de partículas. PS: Profissional de saúde.

**Figura 3:**
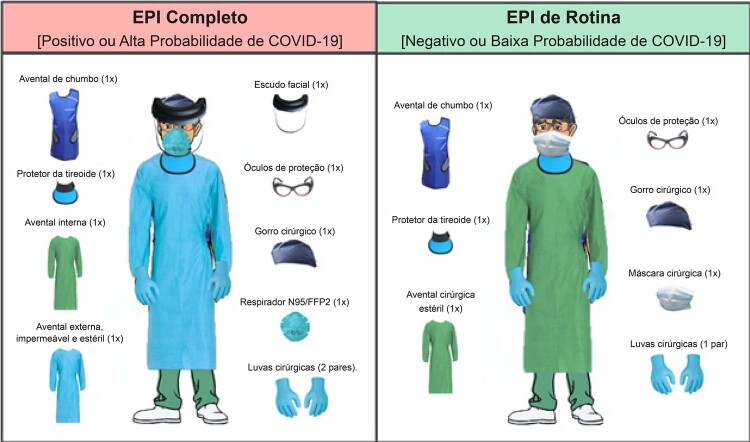
– Modalidades de equipamento de proteção individual. EPI: equipamento de proteção individual.

A Figura 2 mostra os tipos diferentes de máscaras/respiradores disponíveis no nosso instituto de acordo com a eficácia de filtragem de partículas. A Figura 3 mostra as modalidades de EPI para proteção dos PS nos laboratórios de cateterismo cardíaco durante a pandemia de COVID-19.

O EPI completo deve ser usado para os procedimentos em pacientes que apresentaram resultado positivo para SARS-CoV-2 e naqueles classificados como altamente (categoria vermelha) ou moderadamente (categoria amarela) suspeitos de COVID-19. A justificativa para esse EPI é de fornecer proteção contra gotículas respiratórias e garantir a segurança adequada na retirada do EPI, minimizando o contato direto ou indireto com superfícies contaminadas. Esta modalidade de proteção consiste nos itens seguintes:

- Gorro cirúrgico;
- Óculos radiológicos ou óculos de proteção contra respingos;
- Escudo facial;
- Respirador FFP2 ou N95;
- Avental de chumbo e protetor da tireoide;
- Avental cirúrgico interno com manguito nos punhos (não precisa ser estéril);
- Avental cirúrgico externo, impermeável aos fluidos e estéril, com manguito nos punhos;
- Dois pares de luvas cirúrgicas.

O EPI padrão aplica-se a procedimentos em pacientes com resultado negativo para SARS-CoV-2 ou pacientes sem suspeita de COVID. Esta modalidade de EPI consiste nos itens seguintes:

- Gorro cirúrgico;
- Óculos radiológicos ou óculos de proteção contra respingos;
- Máscara cirúrgica;
- Avental de chumbo e protetor da tireoide;
- Avental cirúrgico externo estéril, com manguito nos punhos;
- Um par de luvas cirúrgicas.

**Figura 4:**
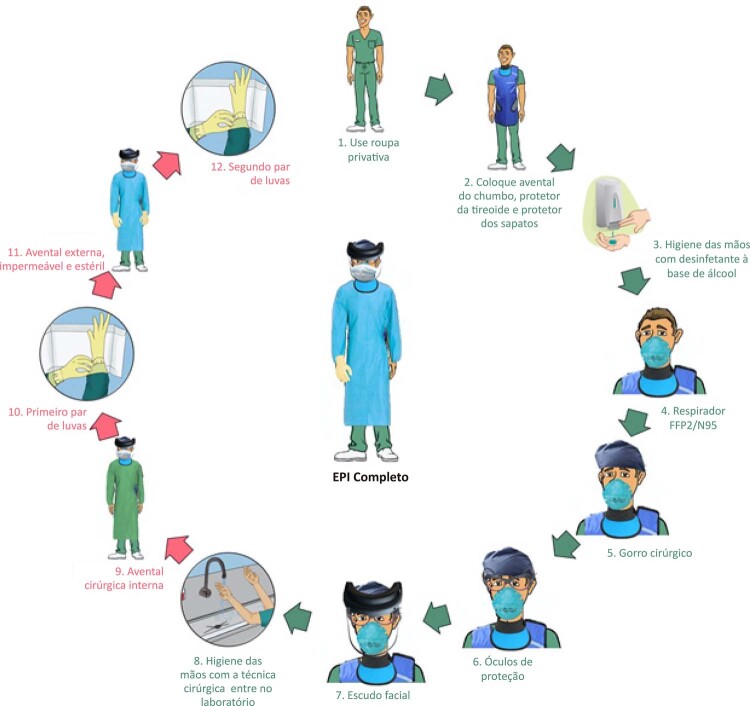
– Sequência de colocação de EPI.

**Sequência de Colocação de EPI ( Figura 4 )**

1. Use a avental cirúrgico;
2. Coloque o avental do chumbo, o protetor da tireoide e protetores de sapatos;
3. Lave as mãos com água e sabão ou desinfetante para as mãos à base de álcool;
4. Coloque o respirador FFP2 ou N95 com a faixa elástica inferior atrás do pescoço e a faixa elástica superior no topo da cabeça. Verifique que o mesmo cobre a boca e o nariz e amarre-o firmemente contra a ponte nasal e as bochechas para minimizar o vazamento de ar da parte superior e dos lados da máscara;
5. Coloque a gorro cirúrgico;
6. Coloque e ajuste os óculos, certificando-se de que não fiquem embaçados durante a respiração;
7. Coloque o escudo facial, ajustando-o firmemente à cabeça;
8. Realize a higiene das mãos com a técnica cirúrgica apropriada;
9. Entre no laboratório de cateterismo e coloque a avental interno;
10. Coloque o primeiro par de luvas. Nesta fase, deve ser realizada a higiene das mãos com a aplicação de uma solução anti-séptica cirúrgica no primeiro par de luvas, caso a avental interno não esteja estéril;
11. Coloque a avental externo, impermeável aos fluidos e estéril;
12. Coloque o segundo par de luvas.

**Figura 5:**
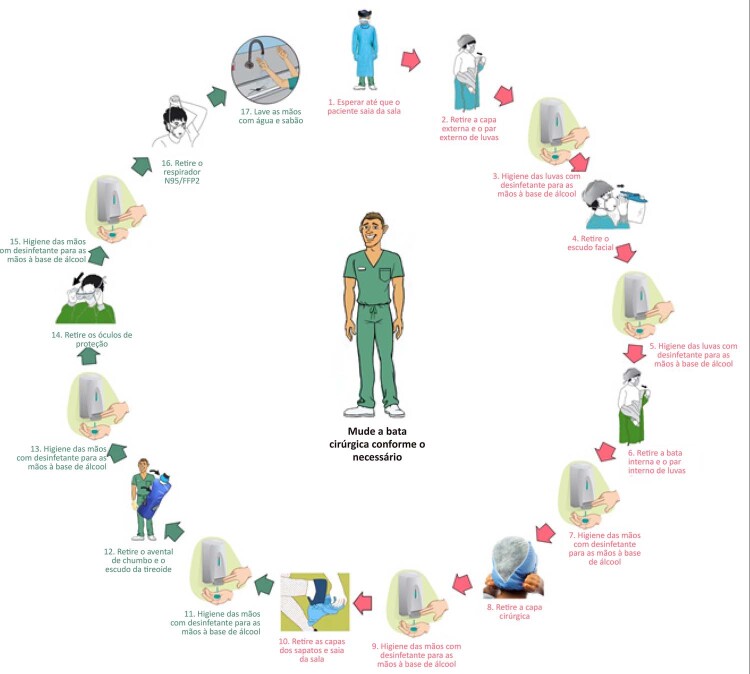
– Sequência de retirada de EPI.

**Sequência de Retirada de EPI ( Figura 5 )**

1. No final do procedimento, pelo menos um membro da equipe médica deve esperar dentro do laboratório até que o paciente saia da sala;
2. Retire a avental externo e o par externo de luvas de dentro para fora. Tenha cuidado para não tocar na superfície externa do avental e das luvas. Descarte-os no recipiente de lixo apropriado;
3. Realize a higiene das mãos com desinfetante para as mãos à base de álcool;
4. Retire o escudo facial, levantando cuidadosamente os lados, evitando tocar no rosto;
5. Realize a higiene das mãos com desinfetante para as mãos à base de álcool;
6. Retire o avental interno e o par interno de luvas completamente e descarte-os no recipiente de lixo apropriado;
7. Realize a higiene das mãos com desinfetante para as mãos à base de álcool;
8. Retire a gorro cirúrgico;
9. Realize a higiene das mãos com desinfetante para as mãos à base de álcool;
10. Retire as protetores dos sapatos e saia da sala;
11. Realize a higiene das mãos com desinfetante para as mãos à base de álcool;
12. Retire o avental de chumbo e o protetor da tireoide;
13. Realize a higiene das mãos com desinfetante para as mãos à base de álcool;
14. Retire os óculos de proteção;
15. Realize a higiene das mãos com desinfetante para as mãos à base de álcool;
16. Retire o respirador FFP2 ou N95 puxando as faixas elásticas sem tocar na face da máscara. Armazene-o em um envelope de papel nomeado e datado para outro uso. O respirador FFP2 ou N95 deve ser descartado se estiver umedecido ou sujo;
17. Lave as mãos com água e sabão;
18. Troque a roupa privativa conforme necessário.

## Apresentações Clínicas

### Infarto do Miocárdio com Supradesnivelamento do Segmento ST

A ICP primária é o padrão de atendimento para pacientes que apresentam IAMCSST e sintomas isquêmicos com duração inferior a 12 horas, desde que possa ser realizada dentro de 120 minutos após o diagnóstico de após o diagnóstico do infarto. ^19^

Devido à indisponibilidade de testes rápidos para SARS-CoV-2, todos os pacientes com IAMCSST estão sendo tratados no laboratório COVID exclusivo, com a equipe usando EPI completo.

Em pacientes com resultado positivo para COVID-19 ou com alta probabilidade (febre, tosse, dispneia, mialgia, contato recente com paciente com COVID-19 ou tomografia computadorizada torácica característica), devem ser equilibrados os benefícios de uma abordagem invasiva e os riscos de exposição da equipe. Nesse sentido, a instituição de uma abordagem fibrinolítica pode ser considerada uma opção para pacientes clinicamente estáveis que apresentam IAMCSST de um território presumivelmente pequeno. A decisão de oferecer fibrinólise como terapia de reperfusão de primeira linha deve ser ponderada individualmente, em discussão conjunta entre o cardiologista do DE e o cardiologista intervencionista. No caso de falha da fibrinólise, a ICP de resgate é realizada no laboratório dedicado para COVID-19.

A lesão miocárdica é uma característica comum da COVID-19, manifestada por elevação dos biomarcadores cardíacos e frequentes anormalidades no ECG e ecocardiograma. Além do infarto do miocárdio devido à ruptura de placa, a lesão miocárdica nestes pacientes também pode ser causada por tempestade de citocinas, lesão hipóxica, espasmo coronariano, microtrombos, lesão endotelial ou vascular direta e perimiocardite, para os quais a fibrinólise não proporcionaria qualquer benefício clínico, aumentaria o risco de sangramento e desencadearia um cateterismo diagnóstico invasivo desnecessário, uma vez que é improvável que a elevação do segmento ST seja resolvida. Uma pequena série de 28 pacientes com COVID-19 apresentando supradesnivelamento do segmento ST mostrou que 39,3% não apresentavam doença arterial coronariana obstrutiva. ^20^

**Figura 6:**
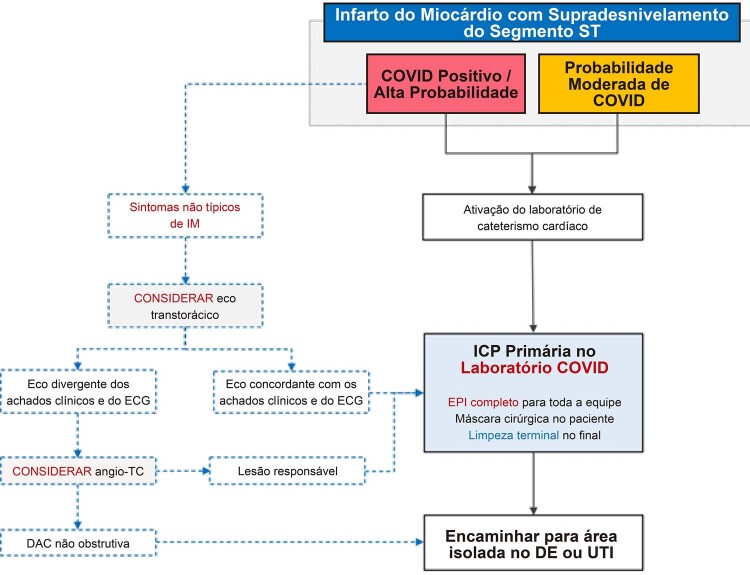
– Fluxo de manejo para pacientes com IAMCSST. DAC: doença arterial coronariana; DE: departamento de emergência; ECG: eletrocardiograma; EPI: equipamento de proteção individual; ICP: intervenção coronária percutânea; IM: infarto do miocárdio; TC: tomografia computadorizada; UTI: unidade de terapia intensiva.

Dessa maneira, está sendo aceita a flexibilização nos padrões de qualidade, como o tempo porta-balão, quando os pacientes chegam ao pronto-socorro com supradesnivelamento do segmento ST, considerando que pode ser necessária avaliação adicional para estabelecer o diagnóstico de infarto agudo do miocárdio verdadeiro e avaliar o estado infeccioso do paciente. Disfunção segmentar no ecocardiograma transtorácico, concordante com as alterações do ECG, podem ser informações úteis. Pode ser considerada a angiotomografia das coronáriasnos casos em que os achados clínicos, eletrocardiográficos e ecocardiográficos são discordantes. Novamente, deverá ocorrer uma discussão conjunta entre o cardiologista do DE e o cardiologista intervencionista para casos individuais, antes de ativar o laboratório de cateterismo cardíaco. A Figura 6 mostra o manejo dos pacientes com IAMCSST.

### Infarto do Miocárdio sem Supradesnivelamento do Segmento ST

A elevação dos biomarcadores cardíacos é comum em pacientes com COVID-19, e está associada a mau prognóstico. ^2 - 4^ Dessa maneira, o teste para COVID-19 deveria idealmente ser realizado antes de encaminhar os pacientes ao laboratório de cateterismo cardíaco. Os pacientes com resultado positivo para COVID-19 ou com alta probabilidade da doença, que não apresentam sinais de SCA de alto risco, devem ser tratados clinicamente. Nestes pacientes, a angiografia coronária invasiva deve ser adiada por pelo menos 15 dias, e sua indicação reconsiderada após a resolução da infecção. Deve-se realizar exames laboratoriais e de imagem para descartar lesões do miocárdio devido à COVID-19 não relacionadas à ruptura típica de placa.

**Figura 7:**
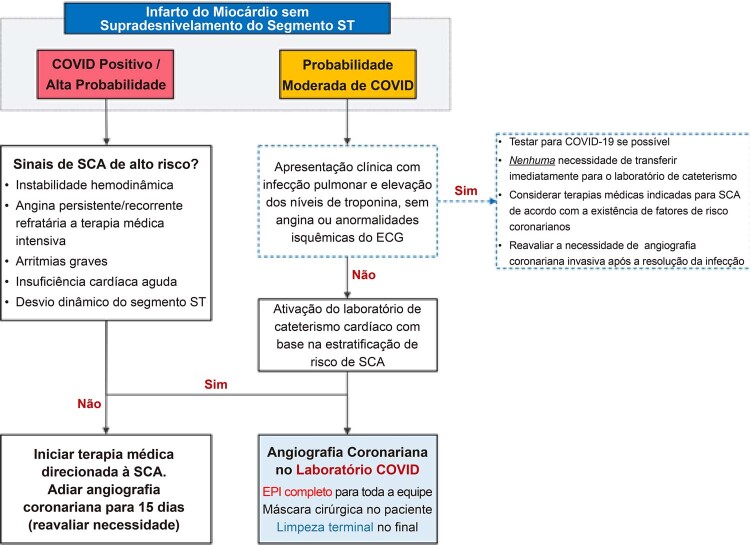
– Fluxo de manejo para pacientes com infarto do miocárdio sem supradesnivelamento do segmento ST. SCA: síndrome coronariana aguda; ECG: eletrocardiograma; EPI: equipamento de proteção individual.

Para os pacientes com SCA de alto risco, a estratificação invasiva deve ser realizada no laboratório COVID-19 com a equipe usando EPI completo ( Figura 7 ).

### Pacientes Encaminhados de Outras Instituições ou Pacientes em Hemodiálise

Na ausência de testes rápidos para COVID-19, esses pacientes serão tratados com as mesmas precauções adotadas para os pacientes com suspeita de COVID-19. O questionário de triagem, aferição da temperatura e a oximetria de pulso são obtidos na chegada desses pacientes. Os procedimentos em pacientes que têm um teste positivo conhecido para SARS-CoV-2 ou que são classificados como altamente prováveis para COVID-19 estão sendo adiados por 15 dias e reavaliados. No caso de um paciente precisar de angiografia coronária urgente, a mesma deve ser realizada no laboratório COVID-19 com a equipe usando EPI completo.

**Figura 8:**
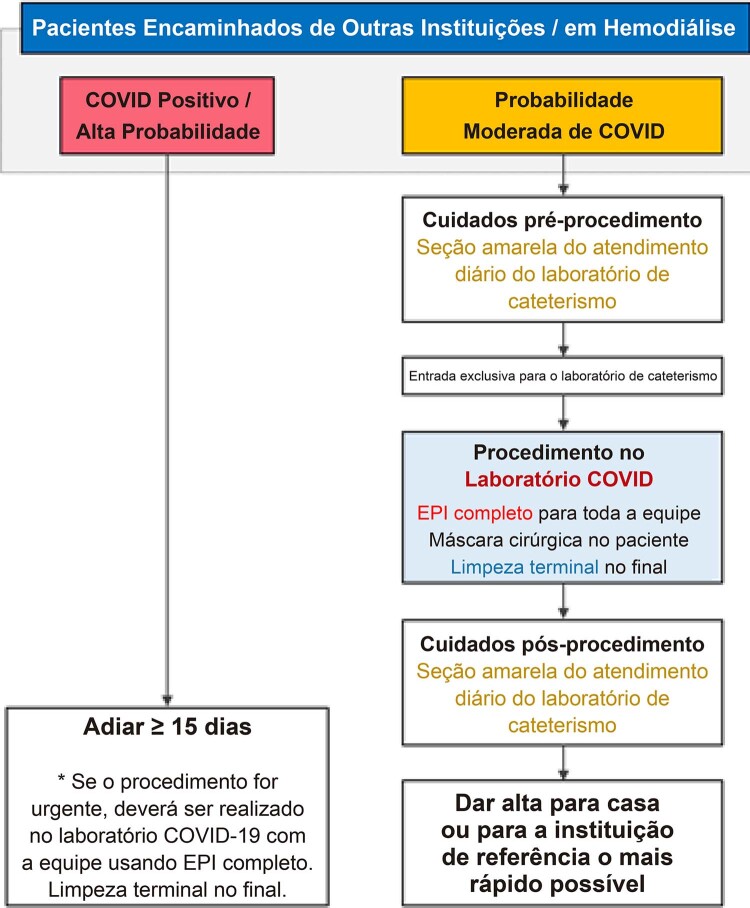
– Fluxo de manejo para pacientes encaminhados de outras instituições ou em hemodiálise. EPI: equipamento de proteção individual.

Os pacientes que não são classificados como de alta probabilidade para COVID-19 recebem cuidados pré e pós-procedimento na seção amarela da unidade de atendimento diário do laboratório de cateterismo; os procedimentos são realizados no laboratório COVID-19 com a equipe usando EPI completo e recebem alta para casa ou para a instituição de referência o mais rápido possível. A Figura 8 mostra o fluxo de manejo para estes pacientes.

### Pacientes Eletivos Vindos de Casa

Desde que o primeiro caso foi relatado no Brasil em 25 de fevereiro, monitoramos a escalada de casos em São Paulo, a demanda de casos suspeitos de COVID-19 chegando ao nosso instituto e a disponibilidade de EPI. De acordo com isso, ajustamos o número de procedimentos eletivos durante este período, a fim de continuar prestando assistência cardiovascular aos necessitados, sem perder o controle dos riscos de transmissão aos PS e os pacientes ou comprometer o suprimento de EPI.

**Figura 9:**
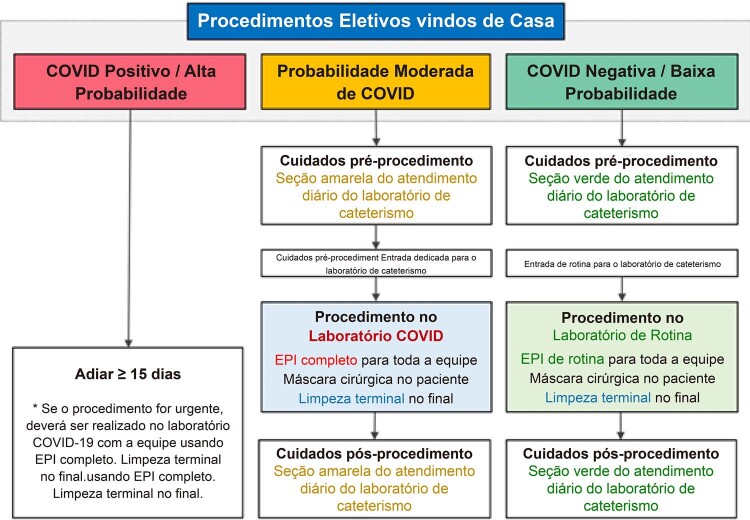
– Fluxo de manejo para pacientes eletivos vindos de casa. EPI: equipamento de proteção individual

No momento da confecção deste manuscrito em 15 de maio, já foram interandos em nosso instituto 136 pacientes com COVID-19 desde o início da epidemia no Brasil. Atualmente, estão internados 59. Durante as últimas duas semanas, a admissão de pacientes suspeitos de COVID-19 aumentou de 2 a 3, no início da epidemia, para 5 a 6 pacientes diariamente. Como resultado, reduzimos o número de procedimentos eletivos, restringindo-os a pacientes altamente sintomáticos e/ou com anatomia de alto risco. São tomadas decisões conjuntas entre cardiologistas e cardiologistas intervencionistas todas as manhãs para discutir a urgência de cada caso eletivo. Os pacientes encaminhados para procedimentos invasivos seguem o processo de triagem descrito anteriormente. A Figura 9 mostra o fluxo de manejo para estes casos.

## Considerações Finais

A atual pandemia de COVID-19 tornou necessário que os hospitais ajustassem suas práticas e vias de atendimento existentes, antecipando um aumento do número de pacientes com COVID-19, mantendo um atendimento de qualidade para pacientes não COVID que apresentam SCA e doença cardíaca isquêmica estável.

Estes ajustes na prática exigem um comportamento dinâmico que deve responder ao padrão epidemiológico de cada região, seus perfis populacionais locais, práticas, logísticas e espaços geográficos, bem como a disponibilidade de EPI.

Tão importante ou talvez até mais importante do que ter EPI disponível é usá-lo corretamente. Uma abordagem multidisciplinar para o desenvolvimento de protocolos internos é desejável, assim como o treinamento frequente e abrangente de toda a equipe, ajustando cada etapa do protocolo conforme necessário.
