# Supplementary material for: Adequação das Práticas do Laboratório de Cateterismo durante a Pandemia de COVID-19: O Protocolo do Instituto Dante Pazzanese de Cardiologia
Source: Arq Bras Cardiol. 2020 Sep 18;115(3):558–68. [Article in Portuguese] doi: 10.36660/abc.20200489 (PMC9363089; doi:10.36660/abc.20200489)
Supplement: Translation

# Translation: Adapted Catheterization Laboratory Practices during the COVID-19 Pandemic: The Instituto Dante Pazzanese de Cardiologia Protocol

**Authors:** Daniel Chamié, Fernanda Oliveira, Sérgio Braga, José Ribamar Costa, Dimytri Alexandre Alvim de Siqueira, Rodolfo Staico, Ricardo Costa, Galo Maldonado, Luiz Fernando Leite Tanajura, Marinella Patrizia Centemero, Áurea Jacob Chaves, Andrea Cláudia Sousa Leão Abizaid, Rafaela Andrade Penalva Freitas, Nancy Toledo Coelho, Louis Nakayama Ohe, Cely Abboud, Fausto Feres

**Affiliations:** 1 Brazil Instituto Dante Pazzanese de Cardiologia, São Paulo, SP – Brazil

**Keywords:** Betacoronavirus/complications, Betacoronavirus/epidemiology, Cardiovascular Diseases/complications, Coronavirus Infections/complications, Practice Guidelines, Test Taking Stills

## Introduction

The global coronavirus disease 2019 (COVID-19) pandemic caused by the novel severe acute respiratory syndrome coronavirus 2 (SARS-CoV-2) virus began in Wuhan, China, in December 2019, and it has affected over 4.4 million people worldwide, with 302,169 deaths as of May 16, 2020. ^1^

Though respiratory symptoms are the most common presentation of COVID-19, cardiac involvement is a prominent feature of this disease, occurring in 20% to 30% of hospitalized patients and contributing to 40% of deaths. ^2-4^ COVID-19-related cardiac involvement has been documented by elevations in cardiac biomarkers, and it often presents with changes in the ST-T segment in the 12-lead electrocardiogram (ECG), for which the catheterization laboratory team is often activated. Furthermore, catheterization laboratory activities must continue for care of non-COVID-19 patients who present with genuine acute coronary syndrome (ACS), ST-elevation myocardial infarction (STEMI), and highly symptomatic stable ischemic heart disease.

Due to the escalation in the number of COVID-19 cases in the city of Sao Paulo, the epicenter of the disease in Brazil, we have reshaped the logistics and practices in the Instituto Dante Pazzanese de Cardiologia cardiac catheterization laboratory, which went into effect at the beginning of April 2020 and will continue for the duration of the pandemic. The goals are to provide optimized care to the population in need of invasive cardiac procedures during the pandemic, with the proper protection to health care professionals (HCP), patients, and their families.

The protocols herein reported represent multidisciplinary and dynamic efforts of the Invasive Cardiology Department at the Instituto Dante Pazzanese de Cardiologia, and they have been validated by the institution’s Infection Control Committee. These practices are subject to change following local epidemiologic status, phase of the epidemic, and availability of personal protective equipment (PPE). These protocols may not apply to other locations with no (or sporadic) COVID-19 cases or to services that treat different population profiles with different logistics and availability of PPE.

### Epidemiology and Transmission of SARS-CoV-2, and Rationale for Reshaping Practices

Current information suggests that the main routes of transmission of SARS-CoV-2 are via respiratory droplets and direct and indirect fomite contact. Exhaled aerosol size depends on the characteristics of the fluid, the force and pressure at the moment of emission, and environmental conditions. Large size particles remain suspended in the air for a short period and settle within 1 meter from the source. Smaller particles evaporate rapidly, while dry residues slowly settle and remain suspended for a variable amount of time. Infectious respiratory aerosols are 1) droplets (respiratory aerosol > 5 μm diameter) and 2) droplet nuclei [dry part of the aerosol (< 5 μm diameter)], which results from the evaporation of coughed or sneezed droplets or from exhaled infectious particles. Usual proximity between people is enough for virus transmission. ^5^ There is a substantial probability that normal speaking causes airborne virus transmission in confined environments. ^6^ Coughing and sneezing can propagate aerosol clouds as far as 7 to 8 meters (23 to 26 feet). ^7^ Droplets may also land on surfaces where the virus can remain viable, serving as a source of contact transmission. SARS-CoV-2 can remain stable in aerosolized form for up to 3 hours, on cardboard materials for 24 hours, and on plastic or metallic surfaces for 3 days. ^8^

The mean incubation period is 5.2 days (95% confidence interval: 4.1 to 7.0 days), with the 95 ^th^ percentile of the distribution at 12.5 days. ^9,10^ The estimated R0 (R naught, basic reproduction number) of SARS-CoV-2 is between 2 and 3, meaning that each person with SARS-CoV-2 is expected to infect 2 to 3 other people in a susceptible population. ^10,11^ The detected viral load is similar in asymptomatic and symptomatic patients with COVID-19, which suggests that the virus can potentially be transmitted from asymptomatic or minimally symptomatic patients. ^12^ Estimates indicate that 86% of all infections were undocumented. Importantly, these were the infection source for 79% of documented cases. ^13^

HCP are at increased risk of infection. A recent report on 138 confirmed COVID-19 cases revealed that 41.3% were acquired in-hospital, 70% of whom were HCP. ^14^ Aerosol generating medical procedures carry greater risk for airborne transmission of the virus to HCP. Among these are tracheal intubation, non-invasive ventilation, tracheotomy, cardiopulmonary resuscitation, manual ventilation before intubation, bronchoscopy, and dental, ophthalmological, and otorhinolaryngological procedures. ^15^

Respiratory viruses are not usually transmitted by blood. ^16^ Thus, for the particular case of HCP in the catheterization laboratory, the higher risk of exposition to SARS-CoV-2 is related to proximity to patients and contact with contaminated fomites, for instance, in surgical drapes, gowns, and equipment.

Ideally, all patients should be tested for SARS-CoV-2 before entering the cardiac catheterization laboratory. Exceptions to this are, perhaps, patients referred for emergency procedures (e.g. STEMI patients), for whom there is no time to wait for test results; these should be treated like patients with confirmed COVID-19. At the moment, tests are not widely available in Brazil, nor are results coming out in a timely manner. Thus, it is intuitive to assume that universal use of full PPE would be desirable. However, PPE shortage is a reality worldwide, and appropriate and rational use of protective equipment is necessary to optimize its availability.

As a result, several strategies have been implemented to provide adequate protection to all HCP and patients without withholding quality patient care.

### Logistic Adjustments Adopted Before Patients Enter the Catheterization Laboratory

- Adjustments in the agenda have been made to minimize waiting times in the reception area of the Invasive Cardiology Department;
- Only one family member or companion per patient is allowed to minimize the number of people circulating in the Invasive Cardiology Department;
- Surgical masks are to be used by all patients and their companions during all times while inside the hospital;
- Medical and nursing staff must use a surgical mask or FFP2/N95 respirators (as appropriate) during contact with patients and hand hygiene after each contact;
- Patients referred from the emergency department (ED) or inpatients referred from other institutions are considered as suspected cases for COVID-19, and full PPE should be used;
- Forehead temperature is to be taken from all patients with non-contact infra-red thermometers upon arrival. Patients with temperatures ≥ 37.0 ^o^ C will have their procedures postponed and will be referred to a suspected COVID-19 area;
- Oxygen saturation is to be taken from all patients with fingertip pulse oximeters upon arrival. Patients with oxygen saturation < 94% will have their procedures postponed and will be referred to a suspected COVID-19 area. Attention must be paid to identification of other medical conditions associated with low oxygen saturation (e.g. decompensated heart failure, chronic lung disease).
- As universal PPE use is not a reality at the moment, we are trying to identify patients who pose a higher risk of infection transmission. Although SARS-CoV-2 transmission can occur in the pre-symptomatic phase, it has been demonstrated that the viral loads in throat swab and sputum samples peaked at around 5 to 6 days after symptom onset. ^17^ Thus, a dedicated questionnaire is being applied to all patients about their symptomatic status and contact with COVID-19 patients. COVID-19 causes a series of constitutional, non-specific symptoms, upper and lower respiratory symptoms, and, less commonly, gastrointestinal symptoms. The three primary symptoms of COVID-19 are fever, cough, and shortness of breath. Other reported symptoms are myalgia, anorexia, malaise, sore throat, nasal congestion, headache, and new loss of taste or smell. Symptoms may appear in as few as 2 days or as long as 14 days after exposure. ^18^ Patients are then risk-stratified for the probability of COVID-19 as shown in Figure 1 .
  **Figure 1:** – Triage questionnaire for patients referred for invasive cardiac procedures. CT: computed tomography. * Characteristic chest CT has been defined as patchy ground-glass opacities with predominant peripheral and basal distribution.
- Full PPE is to be used by all HCP involved in procedures with patients categorized as yellow (moderate COVID probability) or red (high COVID probability).

### Logistic Adjustments in the Routine Practices of the Catheterization Laboratory

As we prepare to care for COVID-19 patients we must ensure that patients without COVID-19 who need invasive cardiovascular care continue to receive care in a safe environment. Therefore, continuous adjustments in the practice of our invasive cardiology facility have been implemented. Key points that drive dynamic changes include the availability of tests for SARS-CoV-2, PPE supply, and health status of the workforce. We provide below a list of the major changes in the practices of our department.

- The 20-bay cardiac catheterization laboratory day care unit is located close to and on the same floor as the catheterization laboratories. This area receives patients coming from home or referred from other hospitals, who are prepared for cardiac catheterizations, percutaneous coronary interventions (PCI), and endovascular and congenital heart disease procedures. It is served by an extensive team of nurses, cardiologists, and first-year cardiology fellows. Post-procedure care is also given in this area. During the COVID-19 pandemic, half of this facility has been dedicated to suspect COVID-19 patients (yellow zone) and the other half to patients without COVID-19 or with a low probability of COVID-19 (green zone). These two zones have been physically separated. The health care team has also been split to cover the two areas independently. Those who care for suspected COVID-19 patients do not look after the COVID negative sector, and vice-versa. Appropriate PPE is available in both sectors.
- Outpatients with suspected COVID-19 and patients coming from the ED, the intensive care unit (ICU), or the COVID area in the hospital enter and exit the cardiac catheterization laboratories via dedicated routes that are different from the ones used by non-COVID patients.
- Of the total six catheterization laboratories, two have been dedicated to COVID-19 positive or suspected patients. In case the number of suspect COVID patients exceeds the number of non-COVID patients, two additional rooms can be converted into COVID labs. We are structured to run four COVID and two non-COVID labs simultaneously.
- The gurneys used to bring suspected or positive COVID patients to the catheterization lab are kept inside the lab to minimize exposure to other areas of the Interventional Department.
- With the aim of reducing the number of circulating personnel and rationing PPE, staffing of COVID labs has been restricted to a minimum, all wearing full PPE.
- The doors of the COVID labs must remain closed during all times.
- None of our catheterization laboratories are equipped with negative pressure systems. Thus, each COVID lab undergoes terminal cleaning following each procedure.

### Protective Equipment for Health Care Professionals

Figure 2 shows the different types of masks/respirators available in our institute according to efficacy of particle filtration. Figure 3 shows the modalities of PPE for protection of HCP in the cardiac catheterization laboratories during the COVID-19 pandemic.

**Figure 2:** – Types of masks/respirators according to their particle filtration efficiency. HCP: Health care professional.

**Figure 3:** – Personal protection equipment modalities. PPE: Personal protection equipment.

Full PPE is to be used for procedures in patients who have tested positive for SARS-CoV-2 and those categorized as highly (red category) or moderately (yellow category) suspect for COVID-19. The rationale for this PPE is to provide protection against respiratory droplets and to ensure adequate safety for PPE doffing, minimizing direct or indirect contact with contaminated surfaces. This modality of protection consists of the following:

- Surgical cap;
- Standard radiological goggles or splash goggles;
- Face shield;
- FFP2 or N95 respirator;
- Lead apron and thyroid shield;
- Internal gown with cuff (does not need to be sterile);
- External, fluid-impermeable and sterile gown with cuff;
- Two pairs of surgical gloves.

Standard PPE applies for procedures in patients who tested negative for SARS-CoV-2 or who have no suspecion of COVID. This modality of PPE consists of the following:

- Surgical cap;
- Standard radiological goggles or splash goggles;
- Surgical mask;
- Lead apron and thyroid shield;
- External sterile gown with cuff;
- One pair of surgical gloves.

#### PPE Donning Sequence ( Figure 4 )

**Figure 4:** – PPE donning sequence.

1. Wear surgical scrubs;
2. Put on the lead apron, thyroid shield, and shoe covers;
3. Wash hands with soap and water or alcohol-based hand sanitizer;
4. Put on the FFP2 or N95 respirator with the lower elastic band behind the neck and the superior elastic band on top of the head. Make sure it covers the mouth and nose, and tie it securely against the nose bridge and cheeks to minimize air leakage through the top and sides of the mask;
5. Put on the surgical cap;
6. Put on and adjust goggles, making sure they do not get fogged up during respiration;
7. Put on the face shield adjusting it firmly to the head;
8. Perform hand hygiene with appropriate surgical technique;
9. Enter the catheterization lab and put on the internal gown;
10. Put on the first pair of gloves. Hand hygiene with the application of a surgical antiseptic solution on the first pair of gloves must be performed at this stage in case the internal gown is not sterile.
11. Put on the external fluid-impermeable sterile gown.
12. Put on the second pair of gloves.

#### PPE Doffing Sequence ( Figure 5 ) 

**Figure 5:** – PPE doffing sequence.

1. At the end of the procedure, at least one member of the medical staff must wait inside the lab until the patient leaves the room;
2. Remove the external gown and the external pair of gloves altogether from inside out. Be careful to not touch the external surface of the gown and gloves. Discard them in the appropriate trash container;
3. Sterilize the internal pair of gloves with alcohol-based hand sanitizer;
4. Remove the face shield by lifting its sides carefully, avoiding touching your face;
5. Sterilize the internal pair of gloves with alcohol-based hand sanitizer;
6. Remove the internal gown and internal pair of gloves altogether and discard them in the appropriate trash container;
7. Hand hygiene with alcohol-based hand sanitizer with alcohol-based hand sanitizer;
8. Remove surgical cap;
9. Hand hygiene with alcohol-based hand sanitizer with alcohol-based hand sanitizer;
10. Remove the shoe covers and exit the room;
11. Hand hygiene with alcohol-based hand sanitizer with alcohol-based hand sanitizer;
12. Remove the lead apron and thyroid shield;
13. Hand hygiene with alcohol-based hand sanitizer with alcohol-based hand sanitizer;
14. Remove protection goggles;
15. Hand hygiene with alcohol-based hand sanitizer with alcohol-based hand sanitizer;
16. Remove the FFP2 or N95 respirator by pulling the elastic bands without touching the face of the mask. Store it in a named and dated paper envelope for another use. The FFP2 or N95 respirator should be discarded if dampened or soiled;
17. Wash hands with soap and water;
18. Change surgical scrubs as necessary.

## Clinical Presentations

### ST-elevation Myocardial Infarction 

Primary PCI is the standard of care for patients presenting with STEMI and ischemic symptoms of less than 12 hours’ duration, provided it can be performed within 120 minutes of STEMI diagnosis. ^19^

Due to the unavailability of quick tests for SARS-CoV-2, all patients with STEMI are being managed in the dedicated COVID lab with staff donning full PPE.

In patients who have tested positive for COVID-19 or who present with a high likelihood (fever, cough, dyspnea, myalgia, recent contact with a COVID-19 patient, or characteristic chest computer tomography scan) the benefits of an invasive approach and the risks of staff exposition must be balanced. On this note, the institution of a fibrinolytic approach can be considered an option for clinically stable patients who present with STEMI of a presumably small territory. The decision to offer fibrinolysis as first-line reperfusion therapy must be weighed on an individual case basis in joint discussion between the ED cardiologist and the interventional cardiologist. In the case of failed fibrinolysis, rescue PCI is performed in the dedicated COVID-19 lab.

Myocardial injury is a common feature of COVID-19, manifested by elevations of cardiac biomarkers, and frequent ECG and echocardiographic abnormalities. Apart from myocardial infarction due to plaque rupture, myocardial injury in these patients can also be due to cytokine storm, hypoxic injury, coronary spasm, microthrombi, direct endothelial or vascular injury, and perimyocarditis, for which fibrinolysis would provide no clinical benefit, increase the bleeding risk, and trigger unnecessary invasive diagnostic catheterization given that the ST-segment elevation is unlikely to resolve. A small series of 28 COVID-19 patients presenting with ST-elevation showed that 39.3% did not have obstructive coronary artery disease. ^20^

Thus, flexibility in quality standards such as the door-to-balloon time is being accepted when patients arrive at the ED with ST-segment elevation, as additional workup may be necessary to establish the diagnosis of genuine acute myocardial infarction and to assess the infectious status of the patient. Regional wall motion abnormality on transthoracic echocardiogram concordant with ECG changes can be useful information. Computed tomography angiography can be considered in cases where the clinical, ECG, and echocardiographic findings are discordant. Again, joint discussion between the ED cardiologist and the interventional cardiologist should take place for individual cases before activating the cardiac catheterization laboratory. The management of STEMI patients is shown in Figure 6 .

**Figure 6:** – Management flow for STEMI patients. MI: myocardial infarction; ECG: electrocardiogram; CT: computed tomography; CAD: coronary artery disease; PPE: personal protection equipment; ED: emergency department; ICU: intensive care unit; PCI: percutaneous coronary intervention.

### Non-ST-elevation Myocardial Infarction 

Elevation of cardiac biomarkers is frequent in patients with COVID-19, and it is associated with poor prognosis. ^2-4^ Thus, testing for COVID-19 should ideally be performed before referring patients to the cardiac catheterization laboratory. Patients who test positive for COVID-19 or patients with high probability of the disease, who do not show signs of high-risk ACS, should be managed clinically. In these patients, invasive coronary angiography should be delayed for at least 15 days, and its indication reconsidered after resolution of the infection. Laboratory and imaging workup should be pursued to discard myocardial injury due to COVID-19 not related to typical ACS plaque rupture.

For patients with high-risk ACS, invasive stratification is to be performed in the dedicated COVID-19 lab with the staff in full PPE ( Figure 7 ).

**Figure 7:** – Management flow for NSTEMI patients. ACS: acute coronary syndrome; ECG: electrocardiogram; PPE: personal protection equipment.

### Patients Referred from Other Institutions or Patients on Hemodialysis 

In the absence of rapid testing for COVID-19, these patients will be managed with the same precautions adopted for suspected COVID-19 patients. The triage questionnaire, forehead temperature, and pulse oximetry are taken from these patients upon arrival. Procedures in patients who have a known positive test for SARS-CoV-2 or who are classified as highly probable for COVID-19 are being postponed for 15 days and reevaluated. In case a patient needs urgent coronary angiography, this is to be performed in the COVID-19 lab with staff using full PPE.

Patients not classified as high probability for COVID-19 receive pre- and post-procedure care at the yellow section of the catheterization laboratory day care; procedures are performed in the COVID-19 lab with staff using full PPE, and they are discharged home or to the referring Institution as soon as possible. Management flow of these patients is shown in Figure 8 .

**Figure 8:** – Management flow for patients referred from other institutions or on hemodialysis. PPE: personal protection equipment.

### Elective Patients Coming from Home 

Since the first case was reported in Brazil on February 25, we have been monitoring the escalation of cases in Sao Paulo, the demand of suspected COVID-19 cases arriving at our institute, and the availability of PPE. We have adjusted the number of elective procedures accordingly over this period in order to keep providing cardiovascular care to those in need without losing track of transmission risks to HCP and patients or compromising the supply of PPE.

As of May 15 ^th^ , a total of 136 patients with COVID-19 had been admitted to our hospital since the start of the epidemic in Brazil. Fifty-nine are currently inpatients. Over the past two weeks, admission of suspected COVID-19 patients rose from 2 to 3 at the start of the epidemic to 5 to 6 patients daily. As a result, we have reduced the number of elective procedures, restricting them to highly symptomatic patients and/or those with high-risk anatomies. Joint decisions between cardiologists and interventional cardiologists are undertaken every morning to discuss the urgency of each elective case. Those referred for invasive procedures follow the triage process previously described. Management flow of these cases is shown in Figure 9 .

**Figure 9:** – Management flow for elective patients coming from home. PPE: personal protection equipment.

## Final Considerations

The current COVID-19 pandemic has made it necessary for hospitals to adjust their practices and existing pathways of care in anticipation of an increase in the number of COVID-19 patients, while maintaining quality care for non-COVID patients presenting with ACS and stable ischemic heart disease.

These adjustments in practice require dynamic behavior that should follow the epidemiologic pattern of each region, their local population profiles, practices, logistics, and geographic space, as well as the availability of PPE.

As important or perhaps even more important than having PPE available is making correct use of it. A multidisciplinary approach to developing internal protocols is desired, as is frequent and comprehensive training of all staff, fine-tuning each step of the protocol as needed.
